# Sacral neuromodulation for the treatment of neurogenic lower urinary tract dysfunction caused by multiple sclerosis: a single-centre prospective series

**DOI:** 10.1186/s12894-015-0102-x

**Published:** 2015-10-23

**Authors:** Daniel S. Engeler, Daniel Meyer, Dominik Abt, Stefanie Müller, Hans-Peter Schmid

**Affiliations:** Department of Urology, Cantonal Hospital St. Gallen, St. Gallen, Switzerland; Department of Neurology, Cantonal Hospital St. Gallen, St. Gallen, Switzerland

**Keywords:** Sacral neuromodulation, Sacral nerve stimulation, Neurologic disease, Lower urinary tract dysfunction, Multiple sclerosis

## Abstract

**Background:**

Sacral neuromodulation is well established in the treatment of refractory, non-neurogenic lower urinary tract dysfunction, but its efficacy and safety in patients with lower urinary tract dysfunction of neurological origin is unclear. Only few case series have been reported for multiple sclerosis. We prospectively evaluated the efficacy and safety of sacral neuromodulation in patients with multiple sclerosis.

**Methods:**

Seventeen patients (13 women, 4 men) treated with sacral neuromodulation for refractory neurogenic lower urinary tract dysfunction caused by multiple sclerosis were prospectively enrolled (2007–2011). Patients had to have stable disease and confirmed neurogenic lower urinary tract dysfunction. Voiding variables, adverse events, and subjective satisfaction were assessed.

**Results:**

Sixteen (94 %) patients had a positive test phase with a >70 % improvement. After implantation of the pulse generator (InterStim II), the improvement in voiding variables persisted. At 3 years, the median voided volume had improved significantly from 125 (range 0 to 350) to 265 ml (range 200 to 350) (*p* < 0.001), the post void residual from 170 (range 0 to 730) to 25 ml (range 0 to 300) (*p* = 0.01), micturition frequency from 12 (range 6 to 20) to 7 (range 4 to 12) (*p* = 0.003), and number of incontinence episodes from 3 (range 0 to 10) to 0 (range 0 to 1) (*p* = 0.006). The median subjective degree of satisfaction was 80 %. Only two patients developed lack of benefit. No major complications occurred.

**Conclusions:**

Chronic sacral neuromodulation promises to be an effective and safe treatment of refractory neurogenic lower urinary tract dysfunction in selected patients with multiple sclerosis.

## Background

Between 50 and 90 % of patients with multiple sclerosis (MS) develop lower urinary tract symptoms (LUTS) in the course of the disease [1, 2]. These symptoms are distressing and have a major impact on quality of life. Treatment of associated lower urinary tract dysfunction (LUTD) is often difficult and may fail. Underlying urodynamic abnormalities include neurogenic detrusor overactivity, detrusor sphincter dyssynergia and detrusor underactivity. These often lead to chronic urinary retention, urge urinary incontinence, and recurrent urinary tract infection. Oral antimuscarinics and, more recently, repeat intradetrusor botulinum toxin type A injections are the most frequent treatment for detrusor overactivity. Many patients need to perform intermittent self-catheterization because of urinary retention, and may eventually need an indwelling catheter or suprapubic cystostomy due to sensorimotor or visual deficits.

Sacral neuromodulation (SNM) has become a well-established approach over the past few years for patients with refractory idiopathic urinary urge incontinence, urgency-frequency syndrome and non-obstructive chronic urinary retention [1–4]. Originally, SNM was not considered an option for neurogenic LUTD, but studies suggest that it is also effective in this these patients [5, 6]. Although LUTS are highly prevalent in patients with MS, only limited case series from 1 to 15 patients treated with SNM have been reported in the literature [7–17]. This low number may be explained partly by the fear of treatment failure due to disease progression.

SNM is minimally invasive and reversible, and may be a valuable treatment option for neurogenic LUTD in MS before resorting to more invasive procedures. The aim of our prospective study was the evaluation of efficacy and safety of sacral neuromodulation in patients with multiple sclerosis based on our experience.

## Methods

### Patient selection

Patients were enrolled prospectively on inclusion in an online registry run by the Swiss Society for Sacral Neuromodulation (SSSNM) founded as a requirement of the Swiss National Department of Health for quality control reasons. All patients had to have MS diagnosed using McDonald criteria [18] and a diagnosis of neurogenic LUTD due to MS confirmed by urodynamic examination. The symptoms could comprise either storage symptoms caused by detrusor overactivity with or without incontinence, or voiding symptoms caused by detrusor underactivity or detrusor sphincter dyssynergia, or both. Exclusion criteria were cognitive impairment precluding the ability to give informed consent for the intervention, progression of MS during the last twelve months, and an expanded disability status scale (EDSS) ≥ 8 preventing independent toilet transfer to enable voiding.

### Assessments at baseline and follow-up

A complete neurourological evaluation was performed at baseline, including a history with clinical examination, assessment of the EDSS, bladder diary, urinalysis, cystoscopy and multichannel urodynamics with electromyogram of the pelvic floor using superficial electrodes (Duet® Encompass™ System, Mediwatch, Rugby, UK). Micturition frequency, voided volume, post void residual and the number of incontinence episodes were recorded as objective variables at baseline and during follow-up, based on a 72-h micturition diary. Incomplete voiding was defined as a repeat post void residual of more than 50 ml. The values of the individual micturition parameters were calculated as means from the diary. Patients rated their grade of satisfaction with the treatment in percent. Clinical data were collected preoperatively after a test phase, and in patients receiving implants, after 6 weeks, and then yearly.

### Test phase

Before permanent implantation, all patients underwent a staged procedure with temporary percutaneous stimulation to assess their response to treatment. This test phase was performed using the definitive tined lead electrode model 3889 (Medtronic Inc., Minneapolis, MN, USA). The procedure was done under local or general anaesthesia with the patient in the prone position. A single intravenous infusion of 2 g cefamandole and 500 mg metronidazole was given as perioperative antibiotic prophylaxis. The test needle and the electrode were positioned under fluoroscopy using the bony landmarks to identify the level of the S3 foramen. After puncture of the S3 foramen on both sides with the test needle, the motor response to electrical stimulation was demonstrated by visible pulling in of the anus to ensure optimal placement of the needle. Monolateral implantation of the quadripolar tined lead electrode was performed on the side with the lower amplitude level for adequate motor response. The lead wire then was tunnelled to the right side in right-handers and to the left side in left-handers. A subcutaneous pocket was created in the posterior gluteal area, and the temporary external wire was connected and tunnelled to the contralateral lumbar side. If the response at both S3 foramina was inadequate, the same procedure was performed on the S4 foramen. Postoperatively, the subacute test phase was conducted with continuous neuromodulation at a sensory amplitude sufficient to induce a light vibratory sensation in the perineum, vagina, bladder or penis. Patients with >70 % improvement in voiding and storage symptoms were regarded as suitable for placement of the permanent pulse generator. If the result was unclear or negative, a second test using an additional contralateral electrode was recommended.

### Implantation of the InterStim II pulse generator

After the test phase, the temporary external wire was cut at the skin level and the InterStim II pulse generator was placed under local or general anaesthesia. A single infusion of 2 g cefamandole was given as perioperative antibiotic prophylaxis. After locating the connecting cluster, the temporary external wire was removed and the InterStim II pulse generator connected, before placing it in the subcutaneous pocket and closing the wound.

### Statistical analysis

GraphPad Prism Version 5.0d (GraphPad Software, Inc., California) was used for the statistical analysis. Outcome data were reported as medians and ranges. The Mann–Whitney and Spearman rank tests were used as appropriate. A *p* value <0.05 was considered statistically significant.

### Ethical approval

Although ethical approval was not sought in advance, the cantonal ethical committee of St. Gallen has evaluated the study (EKSG 15/024) and has confirmed that there is no legal opportunity to approve a study retrospectively. However, upon evaluation of the project, the independent ethical committee has stated that the ethical requirements of this study would have been fulfilled.

## Results

### Patient characteristics at baseline

All 17 patients included prospectively from July 2007 to November 2011 fulfilled the study inclusion and exclusion criteria. The subtype of MS at baseline was relapsing-remitting in 9 (53 %) patients, secondary progressive in 7 (41 %) patients, and primary progressive in 1 (6 %) patient. Most were women (13/17, 76 %). The median age at inclusion was 46.3 years (range 16.9 to 74.6), the median duration of MS was 8 years (range 3 to 46), and the median EDSS at baseline was 5 (range 3 to 7.5).

Incomplete voiding measured by ultrasound or intermittent self-catheterization was present in 16 (94 %) patients. One woman needed an indwelling catheter because of incomplete voiding (>700 ml post void residual) and inability to self-catheterize. The median number of voids per day in the other patients was 12 (range 6 to 20) with a median voided volume of 130 ml (range 70 to 350). The previous urinary tract infection rate was two or more per year in 5 (29 %) patients. The median number of incontinence episodes was 3.5 (range 0 to 10). Five of the patients performed self-catheterization with a median number of 4 per day (range 3 to 6). All patients had failed or not tolerated previous antimuscarinic treatment. One patient had had intradetrusor injection of 200 U onabotulinum toxin A 20 months before developing urinary retention and undergoing temporary cystostomy placement. Three patients also had associated neurogenic faecal incontinence.

Neurogenic LUTD caused by MS was confirmed by urodynamic testing (Table 1). Detrusor overactivity was shown in 15 (88 %) patients, which was phasic in 9, and only terminal in 6 patients. Detrusor overactivity incontinence was present in 9 (53 %) patients. In addition, detrusor sphincter dyssynergia was demonstrated in 7 (41 %) patients.

**Table 1:** Urodynamic variables at baseline

Characteristic	Median value (range)
First urge to void (ml)	107 (38-276)
Strong urge to void (ml)	187 (47-777)
Reflex volume (ml)	80 (8-319)
Maximum cystometric capacity	254 (48-778)
Compliance (ml/cmH_2_O)	10.3 (0.5-80)
Maximum urinary flow (ml/s)	10.3 (0-33.7)
Maximum detrusor pressure during voiding (cmH_2_O)	38 (8-85)
Detrusor pressure at maximum urinary flow (cmH_2_O)	20 (4-200)
Voided volume (ml)	101 (0-414)
Post void residual (ml)	83 (0-778)

### Test phase outcome

Implantation of the tined lead for the test phase was done under local (*N* = 7) or general (*N* = 10) anaesthesia according to patient preference, as described in the methods section. The first test phase was successful in 14 (82 %) patients with an objective improvement of >70 %. One patient had a clearly negative first test phase. This patient refused further testing. Two patients with an equivocal result were tested twice, including the contralateral side. This decision was made after an improvement of less than 70 % during the first test phase. These two patients had better results with bilateral testing. Overall, the test phase was successful in 16 (94 %) patients. The median test duration was 22.3 days (range 3 to 70) and the median period from test start to implantation was 28.5 days (range 3 to 87). No complications occurred during the test phase.

### Implantation

All 16 patients with a positive test phase underwent implantation of the InterStim II pulse generator under local (*N* = 11) or general (*N* = 5) anaesthesia according to patient preference. Two were implanted bilaterally using two InterStim II pulse generators in opposite gluteal positions. Low stimulation amplitudes (<1 V) were achieved in all patients (Table 2). This low level was the result of our attempting to minimize amplitudes and also using subsensory thresholds for stimulation to reduce battery consumption. No perioperative complications occurred.

**Table 2:** Implantation characteristics

Characteristic	Value
Unilateral implantation – N/total N (%)	14/16 (88)
Sacral foramen S3 - N/total N (%)	13/16 (81)^a^
Sacral foramen S4 - N/total N (%)	3/16 (19)^a^
Amplitude – Volt, median (range)	0.58 (0.2-0.9)
Impulse width – μs, median (range)	210 (210-210)
Stimulation frequency – Hertz, median (range)	14 (9-21)
Subsensory stimulation - N/total N (%)	13/16 (81)

^a^one bilaterally

### Follow-up

Fourteen (88 %) patients reached the 3-year follow-up time point. Two had had their device for only 2 years at last follow-up. The maximum follow-up was 6 years. The configuration of electrodes was changed at least once in all 16 patients during follow-up. Several also underwent multiple reprogramming to optimize the treatment effect and reach low amplitudes. The median amplitude used with the InterStim II pulse generator was 0.70 V (range 0.2 to 1.3) after 6 weeks, 0.85 (range 0.35 to 1.5) after 1 year, 0.9 (range 0.6 to 1.2) after 2 years, and 0.85 (range 0.6 to 1.55) after 3 years.

**Fig. 1:**
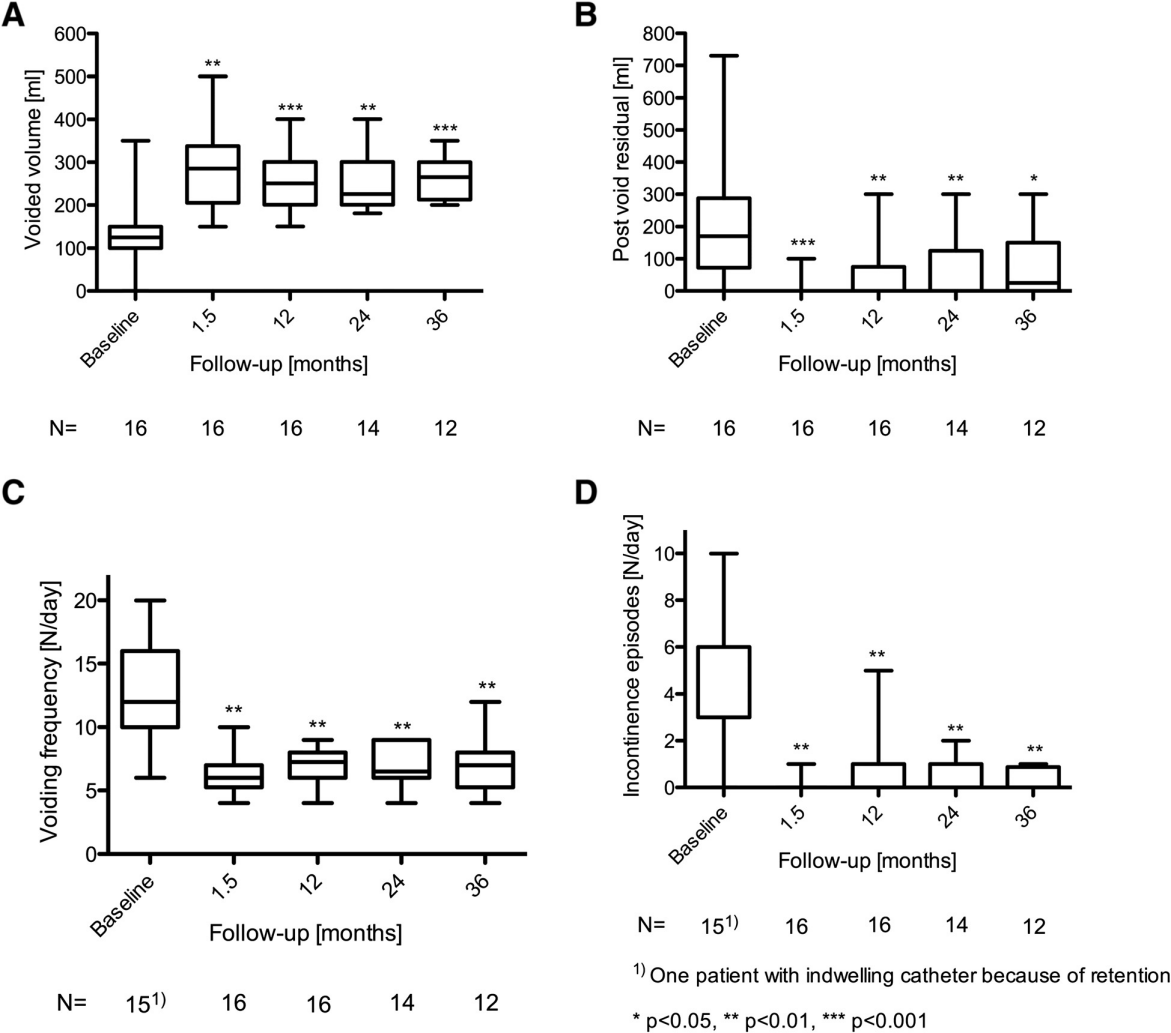
Micturition variables at baseline and follow-up including voided volumes (**a**) post void residual (**b**), voiding frequency (**c**), incontinence episodes (**d**)

Micturition parameters had improved statistically significantly at all measuring times (Fig. 1). At 3 years after implantation, the voided volume had improved significantly from a median of 125 ml (range 0 to 350) before treatment to 265 ml (range 200 to 350) (*p* < 0.001), the median post void residual was reduced from 170 (range 0 to 730) to 25 ml (range 0 to 300) (*p* = 0.01), the median micturition frequency from 12 (range 6 to 20) to 7 (range 4 to 12) (*p* = 0.003), and the median number of incontinence episodes from 3 (range 0 to 10) to 0 (range 0 to 1) (*p* = 0.006).

Subjective assessment of success showed a persistently high degree of satisfaction with a median of 80 % (range 0 to 100) after up to three years. As mentioned above, the effect of the SNM subsided as the disease progressed. Patients with baseline EDSS scores <6 (i.e. able to walk 100 m or more unaided) showed a statistically significantly greater degree of satisfaction after three year’s follow-up than those with EDSS scores ≥6 (*N* = 8 vs. 5, respectively; *p* = 0.025).

### Adverse events

Overall, 5 (31 %) patients underwent revision during follow-up because of problems at the electrode site. There were 2 cases (at 7 and 24 months) of electrode dysfunction with high electrode impedance (>4000 Ohm) for all electrode combinations, loss of sensory response, and clinical impairment of micturition. One patient had a bilateral fracture of the electrodes at 24 months because of a traumatic transverse fracture of the sacrum. Two additional patients had dislocation of their electrode at 18 and 31 months. Revision with exchange of electrodes was successful in all of these cases. One additional patient developed loss of effect during the third year of treatment and decided against reoperation. Another was treated by cystostomy placement because of increasing motor disabilities, and implantation was assessed as a failure because the patient was no longer benefiting. None of the devices were explanted during follow-up. No other complications were observed.

## Discussion

Neurogenic LUTD has a major impact on quality of life in MS patients. The incidence of urinary symptoms increases with disease duration and involvement of the motor system [19]. The anatomic lesions responsible are most often located in the spinal cord [20], although some urinary tract symptoms may also be due to cortical involvement [21].

Treatment options for neurogenic LUTD in MS depend on concomitant motor dysfunction and goal of treatment. Although frequently used and recommended by a UK consensus on the management of the bladder in MS [22] and by the EAU Guidelines on neuro-urology [23] for neurogenic detrusor overactivity, evidence for the use of antimuscarinics is very limited. In MS, disease-specific factors may accentuate LUTS and the side effects of medications, and render symptom management increasingly difficult, including reduced patient compliance. A Cochrane review concluded that anticholinergics (i.e. antimuscarinics) could not be recommended in MS [24]. Other options recommended by the consensus include pelvic floor exercises in patients with mild disability, intermittent self-catheterization if the post void residual is more than 100 ml, and intradetrusor botulinum toxin A injections.

A more recent option is percutaneous tibial nerve stimulation. Clinical improvement in about 80 % of cases has been reported from two prospective non-comparative studies using daily or weekly stimulation schedules [25, 26]. However, treatment effects were assessed over only 3 months.

A minimally invasive approach like SNM that restores the ability to reach the toilet in time and enables complete emptying of the bladder would be a major factor in improving quality of life.

In our patient series treated with SNM, we found a high success rate after failure of conservative treatment options, in agreement with small, previously published series [7–17]. Our test phase was successful (objective improvement >70 %) in a high percentage of patients (94 %). In 6 patients, the test phase was longer than 4 weeks, reflecting our caution in determining the result as positive. However, more prolonged testing did not lead to infectious complications. After implantation, transient deteriorations in effect were generally managed by adaptation of the stimulation parameters and electrode configuration.

A major advantage of SNM over other treatment options is that it not only improves the volume voided, urgency and urge urinary incontinence, but also reduces the post void residual. These effects were achieved early in treatment and remained stable throughout follow-up of up to 3 years.

It is important to note that the degree of subjective satisfaction during follow-up was negatively correlated to the degree of disability at the time of implantation. Not surprisingly, worse motor function at the time of implantation limits the benefits to physical micturition that can be regained. This must be considered when selecting MS patients for SNM.

Our results demonstrate that SNM can be effective even in patients with progressive MS. Even patients with long-standing disease (up to 46 years in our series) can benefit from SNM. Complications we observed were associated with dysfunction or dislocation of electrodes in 5 (31 %) patients, all of which were managed simply by changing the electrodes. Only one patient had complete loss of effect during follow-up, and one other was no longer benefiting from treatment because of motor disabilities.

We included patients prospectively, and follow-up was complete without dropouts over time. A positive treatment effect was evident, despite previous conservative treatment having failed. Nevertheless, the conclusions that can be drawn from this small sample size are limited.

As an implication for research, our findings and those made by others warrant a randomized controlled study to investigate the beneficial effect of SNM in this indication. A Swiss multicentre trial with SNM in patients with neurogenic LUTD is at present under way and we are contributing patients [27].

Thanks to its low associated morbidity, SNM can be considered an option for carefully selected patients with neurogenic LUTD caused by MS. Patients should have failed previous conservative treatment and have stable disease without major motor dysfunction. At best, candidates should be able to walk unaided and should be likely to be able to regain physiological control over micturition. The advent of a number of new disease-modifying medical treatments for relapsing-remitting MS may contribute to prolonging benefit from treatment of their LUTD by SNM.

## Conclusions

Our own mid-term experience and work from others suggests that SNM for refractory neurogenic LUTD due to MS is a good option in carefully selected patients with a high probability of objective and subjective success, including improvement of quality of life. Confirmation in a randomized, controlled trial is needed.

## Abbreviations

EDSS: Expanded disability status scale
LUTD: Lower urinary tract dysfunction
LUTS: Lower urinary tract symptoms
MS: Multiple sclerosis
SNM: Sacral neuromodulation
